# Mixed epithelial and stromal tumor of the kidney with prominent peritumoral adipose tissue: a case report radiologically mimicking angiomyolipoma with epithelial cysts

**DOI:** 10.3389/fonc.2026.1918612

**Published:** 2026-10-02

**Authors:** Chuanchuan Wu, Yang Zhang, Bangwei Che, Jiancheng Zhai, Shenglan Yuan, Linjie Lu, Kai Xiong, Yinqian Li, Kai-Fa Tang

**Affiliations:** 1 The First Clinical Medical College of Guizhou University of Traditional Chinese Medicine, Guiyang, China; 2 Department of Urology & Andrology, The First Affiliated Hospital of Guizhou University of Traditional Chinese Medicine, Guiyang, China

**Keywords:** adipose tissue, angiomyolipoma with epithelial cysts (AMLEC), case report, kidney, mixed epithelial and stromal tumor

## Abstract

Mixed epithelial and stromal tumor of the kidney (MEST) is a rare renal neoplasm with biphasic differentiation comprising epithelial and stromal components. It typically appears as a mixed cystic-solid mass on imaging, and the prognosis is favorable in the vast majority of cases. We report a case of MEST with a prominent fat component on imaging: a 54-year-old woman was admitted with a 1-month history of left flank distension and pain. Contrast-enhanced computed tomography (CT) showed a mixed cystic-solid mass at the lower pole of the left kidney, with a prominent low-attenuation fat-density area surrounding the mass. Preoperatively, the lesion was considered an unusual angiomyolipoma with epithelial cysts (AMLEC). Because the lesion was small, located at the lower pole, and relatively well circumscribed, the patient underwent laparoscopic left partial nephrectomy. Postoperative histopathologic examination confirmed that the left renal mass was benign; however, immunohistochemistry (IHC) demonstrated positivity for EMA and PCK in the epithelial component and for PR and ER in the stromal component, supporting a diagnosis of MEST. The adipose tissue submitted separately with the tumor was located around the tumor and could not be definitively confirmed as an intratumoral component. This case demonstrates that MEST may also exhibit prominent adipose tissue on imaging and illustrates the diagnostic pitfall in which fat-related imaging findings cause MEST to mimic AMLEC, making the early distinction between MEST and cystic change in fat-containing renal tumors challenging.

## Introduction

MEST is composed of admixed epithelial and spindle-cell stromal components with variable morphologies and architectural patterns and shows biphasic differentiation (1–3). It is a rare benign renal tumor (4), occurs mainly in perimenopausal women (5), and most patients present with hematuria, flank discomfort, or urinary tract infection (2, 6); imaging typically shows a mixed cystic-solid mass. MEST with a prominent fat component is extremely rare, with only two case reports previously published (7, 8). Remarkably, in both reports of fat-predominant MEST, the treating physicians made a preoperative diagnosis of an unusual AMLEC (9). In the present case, we likewise initially diagnosed the tumor as an unusual AMLEC, but careful histopathologic examination and immunohistochemical analysis ultimately enabled the correct diagnosis. Although Sireci et al (8) specifically reported a fat-predominant MEST that grossly and radiologically mimicked angiomyolipoma, the significance of the present case lies in its continuous presentation of the preoperative impression of AMLEC, nephron-sparing surgery, and a more comprehensive postoperative pathologic diagnostic process. It also suggests that prominent peritumoral adipose tissue may accentuate this imaging mimicry, making early differentiation of MEST from cystic change in fat-containing renal tumors challenging.

## Case presentation

A 54-year-old woman was admitted with a 1-month history of left flank distension and pain; she reported no hematuria, dysuria, or other discomfort. She had a history of primary hypertension for more than 10 years, with a previous maximum blood pressure of 175/105 mmHg. Her personal and family histories were otherwise unremarkable. Physical examination revealed a palpable left abdominal mass and left costovertebral angle tenderness. After admission, we completed the relevant imaging evaluation. Contrast-enhanced CT showed an approximately 35 × 31 mm, round-to-oval mixed cystic-solid mass at the lower pole of the left kidney. The mass was relatively well circumscribed; its internal cystic hypoattenuating area showed no enhancement, and a large low-attenuation fat-density area surrounded the lesion. No enlarged lymph nodes were identified in the retroperitoneum or adjacent to the great vessels (**Figures 1A, B**). The contrast-enhanced CT findings suggested possible renal cell carcinoma (RCC), without retroperitoneal lymph-node metastasis. However, considering the extensive low-attenuation fat-density area and the cystic lesion, we initially diagnosed an unusual AMLEC. Because the lesion was small, approximately 35 mm in diameter, located at the lower pole of the left kidney, and relatively well circumscribed, nephron-sparing surgery was feasible. Malignancy could not be completely excluded; after discussion, we therefore performed laparoscopic left partial nephrectomy. Intraoperatively, a mass near the lower pole of the left kidney was grossly visible and surrounded by gray-yellow adipose tissue. The mass was sharply demarcated from normal renal tissue and measured 40 × 40 × 20 mm. It was completely resected without complication, with the capsule intact. Histopathologic examination of the resected renal specimen showed a gray-white to gray-yellow solid tumor measuring approximately 35 × 35 × 18 mm, with an intact capsule. Separately submitted gray-yellow adipose tissue measured approximately 70 × 60 × 25 mm and contained no palpable nodules (**Figure 1C**). Importantly, this adipose tissue was submitted separately as peritumoral tissue, and the available tumor sections did not demonstrate definitively intratumoral mature adipose tissue. Microscopically, scattered glandular and cystic epithelial structures of variable size and shape were present. The lining cells were relatively uniform, with focal mild pseudostratification or hobnail morphology. The intervening stroma comprised loosely arranged short fascicles and interlacing bundles of spindle cells with elongated nuclei and evenly distributed chromatin. Cytologic atypia was inconspicuous, mitotic figures were rare, and neither necrosis nor unequivocal infiltrative growth was identified (**Figures 1D, E**). IHC showed that the epithelial component was positive for EMA and PCK; SMA and Desmin were positive; the stromal component was positive for PR, ER, and CD10; the Ki-67 proliferation index was 2%; inhibin-α was negative; and TTF-1 was positive (**Figure 2**). Although the epithelial component in this MEST showed focal TTF-1 positivity, TTF-1 is not entirely specific for pulmonary or thyroid origin; epithelial components of many tumors may show aberrant TTF-1 expression (10). This finding therefore did not indicate metastasis from a pulmonary primary, and TTF-1 is not a core marker for diagnosing MEST; accordingly, it could not be used as the primary diagnostic basis. On the basis of the patient’s symptoms, histopathologic findings, and IHC results, we rejected the initial diagnosis of AMLEC and established a final diagnosis of MEST of the left kidney. The patient recovered well postoperatively, and blood pressure was reasonably controlled at 145-160/85–95 mmHg. She reported no fever, flank pain, hematuria, dysuria, or other symptoms. After treatment including maintenance of electrolyte balance, nutritional support, and wound dressing changes, she was discharged in good condition on postoperative day 3. CT at 6 months showed no local recurrence or distant metastasis. The patient had no flank pain, hematuria, or voiding discomfort. We recommended annual abdominal imaging and renal-function monitoring, as well as active blood-pressure control; because this tumor is rare, a relationship with hypertension cannot be completely excluded. Follow-up in this case remains short (only 6 months), and long-term prognosis requires further observation. The patient’s clinical timeline is presented in **Table 1**.

**Figure 1:**
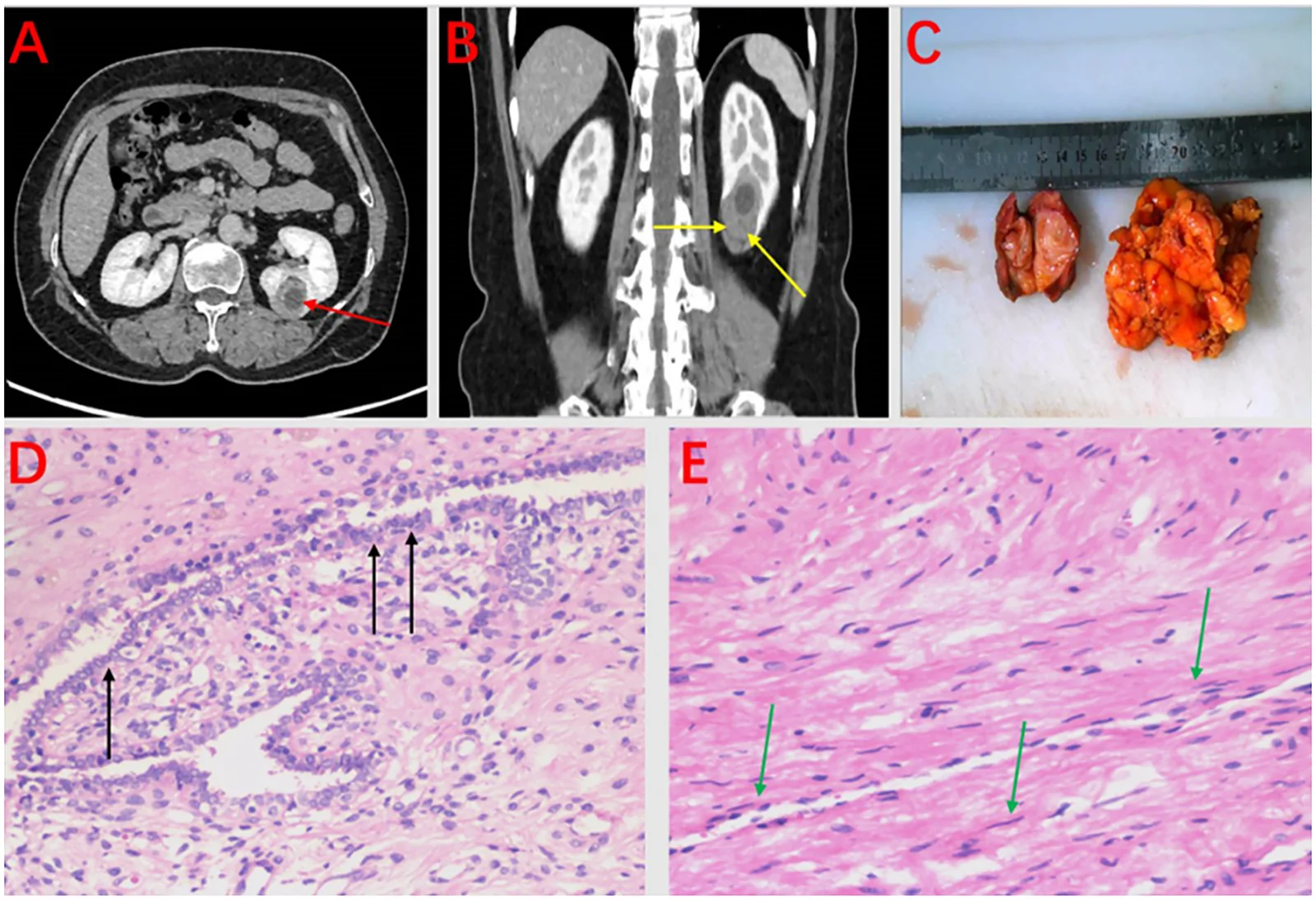
**(A, B)** Contrast-enhanced CT shows a round-to-oval mass measuring approximately 35 × 31 mm at the lower pole of the left kidney. A low-attenuation, non-enhancing cystic area is present within the mass (red arrows), with an extensive surrounding area of low attenuation (yellow arrows). **(C)** The tumor is completely encapsulated. On cut section, it is gray-white to gray-yellow, solid, and of intermediate consistency. A separate aggregate of gray-yellow adipose tissue is also present. **(D)** Hematoxylin and eosin (H&E)-stained section shows scattered irregular tubular and gland-like structures within the epithelial component of the tumor. Some glands exhibit tortuous, branching, and slit-like configurations. The glandular lumina are predominantly lined by a single layer of cuboidal to low columnar epithelial cells, with focal mild pseudostratification or hobnail-like morphology. The epithelial cells show bland cytomorphology with no significant nuclear atypia; no definite mitotic figures or necrosis are identified (black arrow) (original magnification, ×200). **(E)** H&E-stained section shows that the stromal component of the tumor is composed of cytologically bland spindle cells arranged in loose short fascicles and interlacing bundles. The stroma is rich in collagen fibers with focal hyalinization. Cellular atypia is mild, and no definite mitotic figures or necrosis are identified (green arrow) (original magnification, ×200).

**Figure 2:**
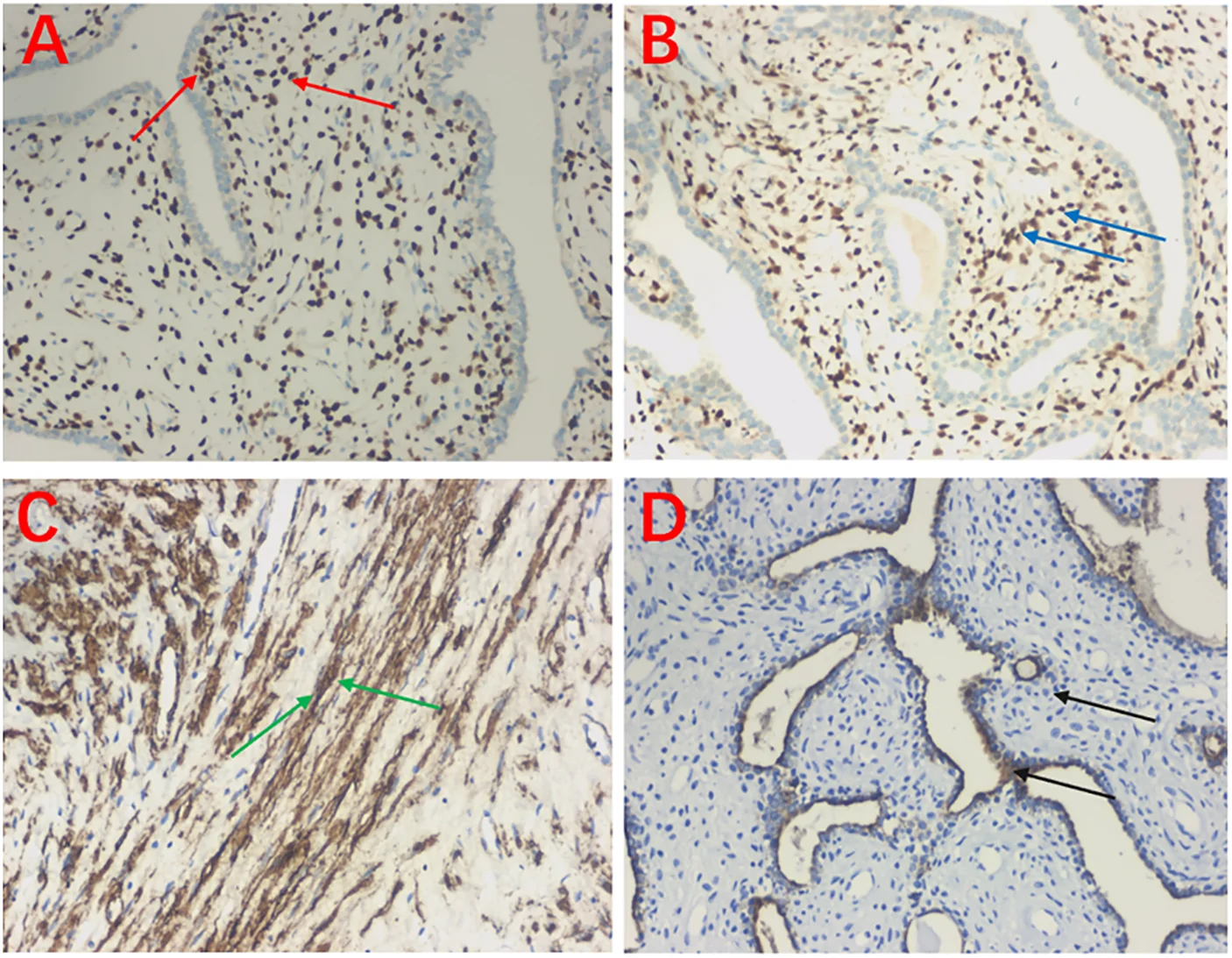
Immunohistochemical staining results. Microscopic examination shows punctate brown-yellow nuclear positivity for ER in the stromal component [**(A)**, red arrow]; punctate brown-yellow nuclear positivity for PR in the stromal component [**(B)**, blue arrow]; diffuse brown-yellow positivity for SMA in the spindle tumor cells [**(C)**, green arrow]; and brown-yellow linear membranous and punctate cytoplasmic positivity for EMA in the epithelial component [**(D)**, black arrow] (original magnification, ×200).

**Table 1:** Clinical timeline.

Time	Clinical event	Main findings or management
1 month before admission	Symptom onset	Left-sided flank distension and pain, without hematuria or dysuria.
At admission	Clinical evaluation	A palpable mass was noted in the left abdomen, with positive left renal angle percussion tenderness. The patient had a 10-year history of hypertension.
During inpatient evaluation	Contrast-enhanced CT	A cystic-solid lesion measuring approximately 35 × 31 mm was identified at the lower pole of the left kidney. The internal cystic component showed no enhancement, and a prominent surrounding fat-density area was observed. Renal cell carcinoma was initially considered; in view of the fat component and cystic architecture, AMLEC was further considered.
During hospitalization	Treatment	Because malignancy could not be excluded and the lesion was relatively small and amenable to nephron-sparing surgery, laparoscopic left partial nephrectomy was performed.
Postoperative pathological evaluation	Diagnosis	The biphasic epithelial and spindle-cell stromal morphology, together with the immunophenotype, supported a diagnosis of MEST. The separately submitted adipose tissue was located around the tumor.
Postoperative day 3	Discharge	Recovery was uneventful, with no fever, flank pain, hematuria, or dysuria. Blood pressure was adequately controlled.
6 months after surgery	Follow-up	No related symptoms were reported. CT showed no evidence of local recurrence or distant metastasis.

## Discussion

The principal educational value of this case is its demonstration of how fat-related imaging findings altered the preoperative assessment of a mixed cystic-solid renal tumor. Fat-predominant MEST mimicking angiomyolipoma has previously been reported (7, 8), but the novelty of the present case lies in its incremental contribution, including the following additional information: the patient had a history of hypertension; the lesion was small and located at the lower pole of the kidney; the imaging differential sequentially included RCC and AMLEC; nephron-sparing surgery was selected; and MEST was ultimately diagnosed on the basis of more comprehensive biphasic histomorphology and the corresponding immunophenotype. Unlike the intratumoral predominance of adipose tissue reported by Sireci et al. (8), the present case definitively documented abundant adipose tissue around the mass, and the available sections were insufficient to establish that the adipose tissue was an intratumoral component. This distinction emphasizes that a perilesional low-attenuation area should not automatically be equated with intratumoral fat on imaging and illustrates the diagnostic ambiguity that complicates early differentiation between adipose tissue-rich MEST and cystic change in fat-containing renal tumors. The patient’s history of hypertension also warrants further consideration: could hypertension and MEST mutually promote disease progression?

MEST is a rare benign renal tumor composed of an admixture of proliferative glandular epithelium and spindle stromal cells (11, 12). Most tumors have a mixed cystic-solid architecture, with interspersed cystic and solid areas, and the cysts may contain clear or hemorrhagic fluid (5). There is a marked sex difference in incidence, with a female-to-male ratio of approximately 6:1, and the tumor most commonly occurs in perimenopausal women (5, 13); the patient in the present case was a 54-year-old woman. Some cases suggest that the development of MEST may be associated with a history of estrogen therapy in women (14). The clinical manifestations of MEST are generally similar across patients. Most patients present with hematuria, flank pain or discomfort, or urinary tract infection, whereas others seek care after a renal mass is detected during a physical examination (2, 6, 15). Our patient presented with distending flank pain and discomfort, consistent with the presentation reported by Tinguria et al. (16). However, these symptoms occur in a wide range of renal diseases, making early diagnosis of MEST on the basis of clinical symptoms difficult.

Similarly, the imaging features of MEST often lack specificity, making early diagnosis difficult. Its imaging appearance may overlap with that of other renal masses, most commonly cystic renal cell carcinoma (CRCC) and cystic nephroma (CN) (17, 18). For example, CN also appears predominantly multicystic on imaging, with mild-to-moderate progressive enhancement of the cyst wall and septa on contrast-enhanced scans (18), potentially complicating the differential diagnosis. In the present case, a hypoattenuating nonenhancing area was seen within the cyst after contrast enhancement, which may help distinguish the lesion from renal tumors with marked internal enhancement. In a previously reported MEST case (16), contrast-enhanced CT showed a predominantly cystic tumor with mild enhancement of the cyst wall and septa in the cortical phase. In our case, CT showed a well-circumscribed mixed cystic-solid lesion at the lower pole of the left kidney with low internal attenuation and no enhancement. These findings indicate that MEST may lack distinctive CT features. In addition, imaging in our patient showed an extensive low-attenuation area around the tumor. Together with the postoperative pathologic finding of gray-yellow adipose tissue in the resected specimen, we speculated that this might be an imaging feature of MEST associated with abundant adipose tissue. In the MEST with abundant adipose tissue reported by Sireci et al. (8), CT likewise demonstrated heterogeneous enhancement and an extensive low-attenuation area. Renal tumors rich in adipose tissue usually include renal angiomyolipoma (AML), renal lipoma, and related entities. Because of the abundant fat associated with the lesion in our case, it was initially misdiagnosed as AMLEC on CT. This case indicates that when imaging demonstrates an extensive low-attenuation area within or around a tumor, clinicians should not focus excessively on the imaging features of fat while overlooking the lesion’s other morphologic characteristics. Such an approach can readily lead to the early misdiagnosis of another fat-rich renal tumor, particularly AMLEC. Therefore, fat-rich MEST may need to be distinguished radiologically at an early stage from cystic change in fat-containing renal tumors.

The definitive diagnosis of MEST usually depends on histopathology and immunohistochemical staining. MEST contains epithelioid cells, smooth muscle cells, adipocytes, and vascular components. Its histopathologic and IHC features may overlap to some extent with those of other renal tumors, making the histopathologic differential diagnosis complex; differential diagnostic features directly relevant to the present case are shown in **Table 2**. AML resembles MEST histopathologically, and both show positivity for SMA and desmin (25, 26). However, MEST contains a higher proportion of epithelial cells, which are often positive for EMA and CK (3), and is negative for human melanoma black 45 (HMB-45) and Melan-A, whereas AML does not show this profile. The immunophenotype of MEST is closely related to its biphasic differentiation. Consistent with the present case, most reported cases (6, 16, 27–29) have shown ER and PR positivity in the stromal component. ER and PR reflect hormone-receptor signaling and serve as key positive markers of MEST. Together with the marked female predominance of this tumor and the history of sex hormone therapy in some patients, high ER/PR expression may indicate the potential for antihormonal therapy in MEST. The epithelial component of MEST is positive for CK and EMA, and the Ki-67 proliferation index is low (2, 6, 16, 27, 29); this pattern also provides important positive markers for diagnosing MEST. However, the epithelial component of some MESTs is positive for paired box 8 (PAX8) (2, 27, 28), necessitating differentiation from cystic change in RCC. In addition, most MESTs express the smooth muscle markers SMA and desmin, which may be related to myofibroblastic differentiation of the tumor (2, 16, 27, 29). Therefore, when MEST is diagnosed by IHC, the relevance and specificity of individual markers must be carefully assessed to achieve a more accurate diagnosis. Finally, at the molecular level, Dicer 1, ribonuclease III (DICER1) gene mutations are major genetic events in the development of CN (30), whereas Vanecek et al. (31) found no somatic DICER1 mutations in MEST of the kidney, which further facilitates differentiation between MEST and CN.

**Table 2:** Differential diagnosis.

Diagnosis	Main morphologic features	Common immunophenotype	Relationship to the present case
MEST (3, 5, 6, 13, 16)	Coexistence of glandular or cystic epithelial components and spindle-cell stroma; the stroma may exhibit ovarian-like or myofibroblastic features.	The epithelial component is positive for EMA and CK; the stromal component is commonly positive for ER, PR, SMA, desmin, and CD10. The Ki-67 proliferation index is usually low.	The biphasic morphology and corresponding immunohistochemical localization support the diagnosis; an intratumoral location of the adipose tissue was not confirmed in the present case.
AMLEC (9, 19)	Epithelial-lined cystic spaces with an underlying dense cambium-like stroma, accompanied by blood vessels, smooth muscle, and variable amounts of adipose tissue.	The stromal component may express ER, PR, CD10, SMA, and desmin; positivity for HMB45 and Melan-A supports the diagnosis.	This entity showed the greatest radiologic similarity to the present case; however, positivity for EMA and CK in the epithelial component is not expressed in AMLEC, and it was therefore excluded.
Multilocular cystic renal neoplasm (20–22)	Predominantly multilocular cystic architecture; the septa may contain ovarian-like stroma, whereas the solid component is usually limited.	The epithelial component may express CK and PAX8; the stromal component may express ER and PR.	It lies within the same tumor family and morphologic spectrum as MEST; the relatively prominent solid spindle-cell stromal component in the present case favors MEST.
Cystic renal cell carcinoma (23, 24)	Neoplastic epithelium is present within the cyst walls or septa, with possible cellular atypia, solid nodules, or invasion.	PAX8 is commonly expressed; expression of CAIX, CK7, and other markers depends on the specific subtype.	This diagnosis could not be excluded preoperatively; however, in the present case, the cells showed bland cytomorphology, mitotic figures were rare, the Ki-67 proliferation index was low, and no definite carcinomatous morphology was identified.

Most MESTs exhibit benign biological behavior. Surgical resection is the preferred treatment, and prognosis is favorable after complete excision; however, rare local recurrences and malignant transformations have been reported (28, 32–34). In the present case, the lesion was small. Although imaging favored a benign process, malignancy could not be completely excluded, providing an indication for surgical exploration. Because the lesion was located at the lower pole of the kidney and was well circumscribed, partial nephrectomy was more appropriate, both providing complete pathologic material and preserving nephrons. However, the absence of recurrence or metastasis at 6 months represents only a short-term outcome and does not permit assessment of long-term recurrence risk. Annual abdominal imaging is planned, together with monitoring of symptoms and renal function.

## Strengths and limitations

The strength of this report is its incremental contribution to the extremely limited literature on fat-predominant MEST. It provides a continuous record of imaging, gross pathology, histology, and IHC findings and clearly presents the diagnostic transition from suspected AMLEC preoperatively to MEST postoperatively, offering educational value for radiologists, urologists, and pathologists. Limitations include: 1. The separately submitted adipose tissue was located predominantly around the tumor, and no serial sampling was available to prove that it was an intratumoral component; 2. the current IHC panel did not include key differential markers such as HMB-45, Melan-A, and PAX8; 3. follow-up was limited to 6 months, precluding assessment of long-term recurrence risk, and a single-case observation cannot be used to estimate the frequency or diagnostic performance of this imaging presentation; 4. the potential relationship between the patient’s history of hypertension and MEST was not investigated further.

## Conclusion

MEST is a rare biphasic renal tumor, and preoperative imaging often lacks specificity. In this case, a mixed cystic-solid lesion and a prominent surrounding fat-density area produced an AMLEC-like appearance; MEST was ultimately diagnosed on the basis of the histomorphology of the epithelial and spindle-cell stromal components and their corresponding immunophenotypes. Fat-predominant MEST has previously been reported, but the strength of this case lies in its documentation of the complete diagnostic transition, nephron-sparing treatment, and a more comprehensive pathologic diagnostic process. A limitation is that the available sections confirm only that the adipose tissue was peritumoral and cannot definitively establish an intratumoral fat component. Nevertheless, this case indicates that when a similar lesion is encountered, imaging localization, serial sampling, and histologic correlation should be used to determine the relationship between the adipose tissue and the tumor, and cystic change in fat-containing renal tumors should be considered in the differential diagnosis.

## Patient perspective and follow-up

The patient was satisfied with the diagnostic and treatment process and with her postoperative recovery. At the 6-month follow-up, she had no flank pain, hematuria, or voiding discomfort, and CT showed no local recurrence or distant metastasis. Although MEST is usually benign, rare recurrences and malignant transformations have been reported. Annual abdominal imaging was therefore recommended, together with long-term follow-up based on symptoms and renal function.

## Data Availability

The original contributions presented in the study are included in the article/supplementary material. Further inquiries can be directed to the corresponding author.

## References

[1] Picken MM Bova D Pins MR Quek ML . Mixed epithelial and stromal tumor of the kidney with extension into inferior vena cava: Case report and discussion of adult biphasic cystic renal lesions and the significance of vascular involvement. Case Rep Pathol. (2018) 2018:8234295. doi: 10.1155/2018/8234295 30364101 PMC6188733

[2] Bakavičius A Barisienė M Snicorius M Valančienė D Dasevičius D Žalimas A . Malignant mixed epithelial and stromal tumour of the kidney: A case report and a literature review. Acta Med Lituanica. (2018) 25:31–7. doi: 10.6001/actamedica.v25i1.3701 29928155 PMC6008004

[3] Kalinowski P Kalinowski T Kucharz J Kamecki H Adamowicz B Sikora K . Mixed epithelial and stromal tumor of the kidney: A case report. Oncol Lett. (2023) 25:25. doi: 10.3892/ol.2022.13611 36478902 PMC9713829

[4] Karasavvidou F Mitrakas L Tzika S Zachos I Anagnostou A Tzortzis V . Mixed epithelial and stromal tumor-adult cystic nephroma of the kidney: A case report with immunohistochemical analysis. J Surg Case Rep. (2022) 2022:rjac387. doi: 10.1093/jscr/rjac387 36105648 PMC9467513

[5] Arriola AGP Taylor BL Ma S Malkowicz SB Lal P . Malignant mixed epithelial and stromal tumor of the kidney with 2 simultaneous renal carcinomas in a male patient: Case report and review of the literature. Int J Surg Pathol. (2018) 26:56–63. doi: 10.1177/1066896917720032 28697675

[6] Dharwadkar A Gore C Bhuibhar G Viswanathan V . Unmasking the mystery of renal neoplasm in a perimenopausal woman: A case report. Cureus. (2024) 16:e56970. doi: 10.7759/cureus.56970 38665735 PMC11044075

[7] Takeuchi Y Sekido N Sawada Y Kuroda K Oharaseki T . A case of mixed epithelial and stromal tumor (MEST) of the kidney monitored as angiomyolipoma (AML) : a case report. Hinyokika Kiyo. (2013) 59:641–5. 24262704

[8] Sireci AN Rodriguez R Swierczynski SL Netto GJ Argani P . Fat-predominant mixed epithelial stromal tumor (MEST): Report of a unique case mimicking angiomyolipoma. Int J Surg Pathol. (2008) 16:73–7. doi: 10.1177/1066896907304523 18203791

[9] Fine SW Reuter VE Epstein JI Argani P . Angiomyolipoma with epithelial cysts (AMLEC): A distinct cystic variant of angiomyolipoma. Am J Surg Pathol. (2006) 30:593–9. doi: 10.1097/01.pas.0000194298.19839.b4 16699313

[10] Möller K Gulzar T Lennartz M Viehweger F Kluth M Hube-Magg C . TTF-1 is a highly sensitive but not fully specific marker for pulmonary and thyroidal cancer: A tissue microarray study evaluating more than 17,000 tumors from 152 different tumor entities. Virchows Archiv: Int J Pathol. (2024) 485:815–28. doi: 10.1007/s00428-024-03926-1 39377914 PMC11564378

[11] M YH Fayaz SN Ganesh SR Lazim NM Tp R Fathima R . Mixed epithelial and stromal tumor of the kidney: Clinicopathologic features and surgical outcomes in four patients. Cureus. (2025) 17:e77562. doi: 10.7759/cureus.77562 39958071 PMC11830114

[12] Jiménez Domínguez AC Álvarez Lucena AJG Vila Braña P Vázquez Alonso F . Mixed epithelial and stromal kidney tumor with sarcomatous transformation. A case report. Actas Urologicas Espanolas. (2025) 50(1):501692. doi: 10.1016/j.acuroe.2025.501692 39947296

[13] Mai KT Elkeilani A Veinot JP . Mixed epithelial and stromal tumour (MEST) of the kidney: Report of 14 cases with male and PEComatous variants and proposed histopathogenesis. Pathology. (2007) 39:235–40. doi: 10.1080/00313020701230799 17454754

[14] Mohanty SK Parwani AV . Mixed epithelial and stromal tumors of the kidney: An overview. Arch Pathol Lab Med. (2009) 133:1483–6. doi: 10.5858/133.9.1483 19722760

[15] Lane BR Campbell SC Remer EM Fergany AF Williams SB Novick AC . Adult cystic nephroma and mixed epithelial and stromal tumor of the kidney: Clinical, radiographic, and pathologic characteristics. Urology. (2008) 71:1142–8. doi: 10.1016/j.urology.2007.11.106 18313107

[16] Tinguria M Chorneyko K . Mixed epithelial and stromal tumor: A rare renal neoplasm-case report with clinicopathologic features and review of the literature. Case Rep Pathol. (2023) 2023:528377. doi: 10.1155/2023/3528377 36644654 PMC9833914

[17] World Health Organization . Urinary and Male Genital Tumours Vol. 8. . Lyon: International Agency for Research on Cancer (2022). Available online at: https://tumourclassification.iarc.who.int (Accessed December 2025).

[18] Fang YW Song HC Sun N Zhang WP . Non-Wilms' renal tumors in children: Experience with 139 cases treated at a single center. BMC Urol. (2022) 22:89. doi: 10.1186/s12894-022-01042-3 35733141 PMC9219123

[19] Guan H Zhang L Zhang Q Qi W Xie S Hou J . Primary angiosarcoma arising in an angiomyolipoma of the kidney: Case report and literature review. Diagn Pathol. (2018) 13:53. doi: 10.1186/s13000-018-0730-z 30111336 PMC6094454

[20] Peng Y Jia L Sun N Li J Fu L Zeng J . Assessment of cystic renal masses in children: Comparison of multislice computed tomography and ultrasound imaging using the Bosniak classification system. Eur J Radiol. (2010) 75:287–92. doi: 10.1016/j.ejrad.2010.05.035 20594787

[21] Xu B Mi Y Zhou LQ Jin J Zhang Q Chen GF . Laparoscopic partial nephrectomy for multilocular cystic renal cell carcinoma: A potential gold standard treatment with excellent perioperative outcomes. World J Surg Oncol. (2014) 12:111. doi: 10.1186/1477-7819-12-111 24754899 PMC4000319

[22] Kurian JJ Jehangir S Korula A . Multiloculated cystic renal tumors of childhood: Has the final word been spoken. J Indian Assoc Pediatr Surgeons. (2018) 23:22–6. doi: 10.4103/jiaps.JIAPS_224_16 29386760 PMC5772090

[23] Kirkali Z Canda AE . Open partial nephrectomy in the management of small renal masses. Adv Urol. (2008) 2008:309760. doi: 10.1155/2008/309760 18645618 PMC2467459

[24] Zhu Q Ling J Ye J Zhu W Wu J Chen W . CT and MRI findings of cystic renal cell carcinoma: Comparison with cystic collecting duct carcinoma. Cancer Imaging: Off Publ Int Cancer Imaging Soc. (2021) 21:52. doi: 10.1186/s40644-021-00419-1 34493335 PMC8422719

[25] Varshney B Vishwajeet V Madduri V Chaudhary GR Elhence PA . Renal angiomyolipoma with epithelial cyst. Autopsy Case Rep. (2021) 11:e2021308. doi: 10.4322/acr.2021.308 34458176 PMC8387069

[26] Fejes Z Sánta F Jenei A Király IE Varga L Kuthi L . Angiomyolipoma of the kidney-clinicopathological analysis of 52 cases. Pathol Oncol Research: POR. (2023) 28:1610831. doi: 10.3389/pore.2022.1610831 36699622 PMC9868137

[27] Nikolić G Simić L Životić M Stefanović J Mioljević A Ćuković L . Myopericytoma/myofibroma as a stromal component of mixed epithelial and stromal tumor of the kidney: A case report. Exp Ther Med. (2024) 28:322. doi: 10.3892/etm.2024.12610 38939172 PMC11208761

[28] Dan C Sahai A Suri P Singh J Trehan RS . Mixed epithelial stromal tumor of the kidney with a mesenteric lymph node: A case report and literature review. Cureus. (2023) 15:e46058. doi: 10.7759/cureus.46058 37900393 PMC10605561

[29] Gokden N Dawson K Lindberg M . Malignant rhabdoid tumor arising in a mixed epithelial, stromal tumor of kidney: Report of a male case, review of the literature. Pathol Res Pract. (2020) 216:153151. doi: 10.1016/j.prp.2020.153151 32853941

[30] Schultze-Florey RE Graf N Vorwerk P Koscielniak E Schneider DT Kratz CP . DICER1 syndrome: A new cancer syndrome. Klinische Padiatrie. (2013) 225:177–8. doi: 10.1055/s-0033-1337976 23625684

[31] Vanecek T Pivovarcikova K Pitra T Peckova K Rotterova P Daum O . Mixed epithelial and stromal tumor of the kidney: Mutation analysis of the DICER 1 gene in 29 cases. Appl Immunohistochemistry Mol Morphology: AIMM. (2017) 25:117–21. doi: 10.1097/PAI.0000000000000262 26509905

[32] Sun BL Abern M Garzon S Setty S . Cystic nephroma/mixed epithelial stromal tumor: A benign neoplasm with potential for recurrence. Int J Surg Pathol. (2015) 23:238–42. doi: 10.1177/1066896914563391 25525149

[33] Mohd Zam NA Lau WK Yip SK Cheng CW Tan PH . Mixed epithelial and stromal tumour (MEST) of the kidney: A clinicopathological report of three cases. Pathology. (2009) 41:403–6. doi: 10.1080/00313020902886944 19404861

[34] Holkar PS Jain T Kavishwar V Pandya JS . Metastasis in mixed epithelial stromal tumour of the kidney: A rare presentation. BMJ Case Rep. (2019) 12:e229293. doi: 10.1136/bcr-2019-229293 31570342 PMC6768369

